# Physical education movement and comprehensive health quality intervention under the background of artificial intelligence

**DOI:** 10.3389/fpubh.2022.947731

**Published:** 2022-09-16

**Authors:** Bo Zhang, Hao Jin, Xiaojing Duan

**Affiliations:** ^1^Department of Physical Education and Teaching, Hebei Finance University, Baoding, China; ^2^Department of Sports Work, Hebei Agricultural University, Baoding, China; ^3^Department of Functional Ultrasound, Affiliated Hospital of Hebei University, Baoding, China

**Keywords:** artificial intelligence, physical education, comprehensive health quality, virtual simulation technology, experimental teaching

## Abstract

The application of artificial intelligence has realized the transformation of people's production and lifestyle, and also promoted the progress of physical education and comprehensive health quality. The application of artificial intelligence in the current physical education movement is increasing. By utilizing its advanced method of virtual simulation technology, the purpose of this paper is to realize the interventional research on the physical education movement and comprehensive health quality in the environment of artificial intelligence. This paper proposes to use the virtual simulation technology and Kinect algorithm in artificial intelligence to design the virtual sports simulation teaching mode. The functional module design part where the Kinect algorithm helps the teaching of virtual sports simulation experiments, which is helpful to analyze and solve the objective system imbalance and ecological imbalance in online physical education teaching. By using the principles and rules of the Mean Shift image segmentation algorithm for reference, the investigation and research on the comprehensive health quality of students are carried out, so as to realize the ecologicalization of the virtual sports school. In the investigation and research on the comprehensive quality of students, the results show that the overall quality of these students who has reached the level of qualified or unqualified is accounting for about 30% of the total number. It is worth noting that in terms of scientific and cultural quality, only 43.34% of all students have excellent grades. It can be seen that the important training goal of current school research is how to use reasonable and effective methods and strategies to improve students' scientific and cultural level, and improve students' other comprehensive scores at the same time.

## Introduction

The virtual simulation experiment teaching in artificial intelligence is the need of physical education. However, after investigation, it was found that there were some problems such as different configuration levels and insufficient quantity of the current sports experimental equipment, which resulting in some experiments that could not meet the needs of standard teaching classes. Therefore, physical education needs the assistance of modern high-tech to meet the needs of physical education experimental teaching. The influence of virtual simulation experiment teaching in the field of teaching comes from the vigorous development of contemporary information technology. Its construction and development is an important way to comply with Chinese policies, respond to industry needs, and promote the development of physical education. First of all, virtual simulation experiment teaching is the need of the development of the times. Throughout the development of teaching form, it has experienced a development path from plane to three-dimensional, single to complex, and simple to depth. However, subject to the influence of various objective factors, the leap forward of experimental teaching to this process is restricted, which in turn affects the teaching effect of experimental teaching application. Therefore, virtual simulation experiment teaching is in line with the needs of the development of the times.

The research on physical education and comprehensive health quality has always been a hot topic. It is a relatively new field to study the two together. From the investigation, it can be found that the current sports experimental equipment has problems such as different levels of configuration and insufficient quantity, which results in some experiments failing to meet the standards. And there are many choices to carry out related research. Among them, Evseev et al., aimed to demonstrate the improvement of the All-Russian sports program “Preparing People with Disabilities and Limited Health.” It focused on the basic physical fitness of PHL and its ultimate value through a healthy lifestyle (1). Silverman proposed to discuss measurement issues in attitude research, which focusing on the question of score reliability. Finally, the results of the research on the attitudes of PE students and teachers were reviewed (2). Han et al., used edge computing technology to design a balanced sports development framework (3). Zhao described the role and significance of scientific training in developing competitive sports in colleges and universities, and put forward suggestions on the various procedures and implementation of training (4). The goal of Mandra et al., was to develop and justify complex treatment strategies and the prevention of hard tooth tissue disease based on clinical and laboratory studies of the state of hard tooth tissue in athletes (5). Although the above research has promoted the development of physical education and comprehensive quality of health to a certain extent. However, it is only in the theoretical stage at present, and has no practicality.

In order to promote the interventional research on physical education and comprehensive health quality, the traditional physical education model needs to be improved. As the current mainstream science, artificial intelligence has applications in many fields, so many scholars' researches on artificial intelligence are as follows. Among them, Dye et al., proposed that artificial intelligence applications in earth sciences were becoming more common, but there were still many challenges in applying established techniques to earth science datasets (6). Musulin et al., discussed the applicability of machine learning (ML) and evolutionary computation (EC) methods focused on regressing the epidemiological curve of COVID-19, and outlined the usefulness of existing models in specific domains (7). Hariharan et al., applied artificial intelligence system to control the maximum power of photovoltaic system with maximum response time. In this perturbation and observation algorithm, the maximum power in the photovoltaic system was achieved by a fuzzy logic system (8). Based on the model of Software Engineering, Saini et al., provided various forms of modeling for capturing structural, behavioral, configuration, and intent aspects of software systems. One of the most widely used models in the early stages of requirements analysis or design was the domain model (9). Bagaria and Tiwari proposed that artificial intelligence (AI)-enabled robots might become more advanced and overcome existing challenges such as cost, training, and improved performance based on feedback provided by surgeons (10). However, the application fields of artificial intelligence mentioned above refer to the use of artificial intelligence in some researches on the human body, which do not represent the intervention research of artificial intelligence in physical education and comprehensive health quality. Moreover, these methods still have the problems of ineffectiveness and high cost.

This paper uses the Kinect algorithm and virtual simulation technology in artificial intelligence. Through the research and model design of the current physical education teaching, and the use of Internet technology to investigate and analyze the current comprehensive quality of health, the results show that: at present, the students' needs for the teaching content of virtual simulation experiments are sports physiology knowledge, sports technology analysis and diagnosis, and sports rehabilitation knowledge. The selection rates are 79.71, 71.39, and 67.82% respectively. The selection rate of sports anatomy knowledge and sports biomechanics is slightly lower than the first three, which is 63.44 and 51.22% respectively. It shows that the subjects of the survey are strongly biased toward sports physiology, sports technology analysis and diagnosis, and sports rehabilitation knowledge in the teaching content of virtual simulation experiments, but there is also a high demand for sports anatomy and sports biomechanics. It can be seen from the proportion of the relevant classes in the various quality levels of the surveyed students that the overall quality of these students reaches the pass or fail level, which is about 30% of the total number of students. It is worth noting that the scientific and cultural quality, the proportion of good grades among all students is only 43.34%. From this, it can be seen that the important training goal of current school research is how to use artificial intelligence methods and strategies to enhance students' scientific and cultural level and improve students' comprehensive quality in other aspects at the same time. However, the current research on physical education under artificial intelligence still does not get rid of the definition and thinking of traditional physical education sports and health comprehensive quality. There is also a lack of in-depth analysis and discussion of the functionality of artificial intelligence, which hinders the high integration and advantages of artificial intelligence technology and physical education.

The innovation of this paper is based on the construction of virtual simulation experiment teaching project. First of all, it is conducive to the timely update of the corresponding experimental teaching content and equipment, and the richness of the experimental teaching course content. Secondly, it is conducive to improve the safety of the experiment, which makes the experimental process safe and reliable. And the experimental practice can be repeated many times, which helps students to improve their practical skills more easily. Finally, it is helpful to solve the dilemma such as the shortage of experimental resources and the easily damaged experimental equipment and instruments, and alleviate the difficulties in the development of experimental teaching.

## Design of virtual sports simulation teaching mode based on artificial intelligence

### Physical education experiment teaching

**Figure 1:**
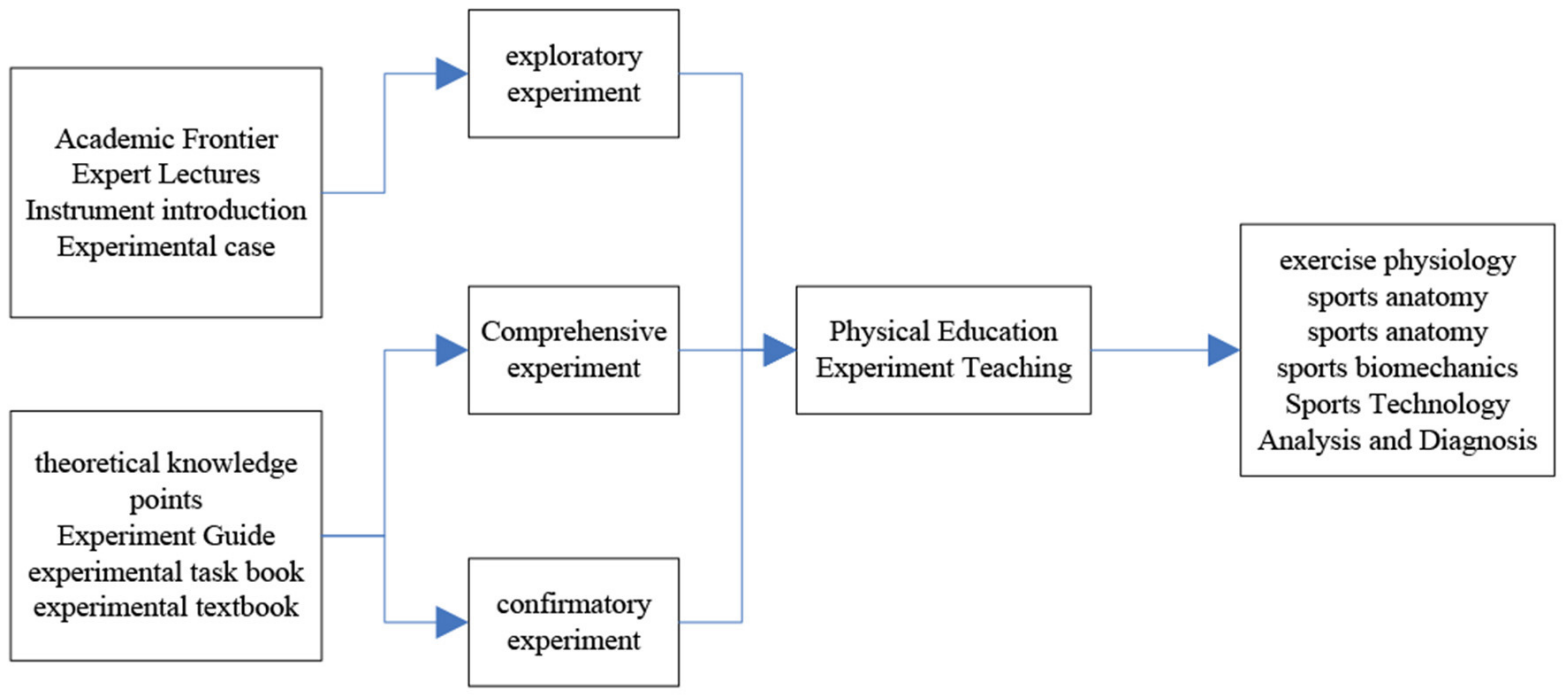
Sports experimental model.

Experimental teaching is a teaching activity that integrates knowledge, ability and practice. It has the same teaching status as theoretical teaching, and has become an indispensable part of colleges and universities to cultivate students' practical ability, creativity and exploration ability. Chinese scholars have reached a consensus on the important role of experimental teaching in teaching, scientific research and social services in colleges and universities (11). Physical education experimental teaching is a teaching activity that promotes physical education students to master solid knowledge and cultivates physical education students' practical ability and decision-making ability to solve human scientific problems. The model is shown in Figure 1.

As can be seen from Figure 1, with the increasing social demands, the current society requires sports talents not only to have a solid scientific foundation and the ability to master cutting-edge knowledge in the field of sports, but also to require students have solid practical hands-on application ability to solve complex human science problems in real-world environments. Therefore, physical education experimental teaching is the key component of training compound sports talents in higher physical education colleges (12).

### Smart education

**Figure 2:**
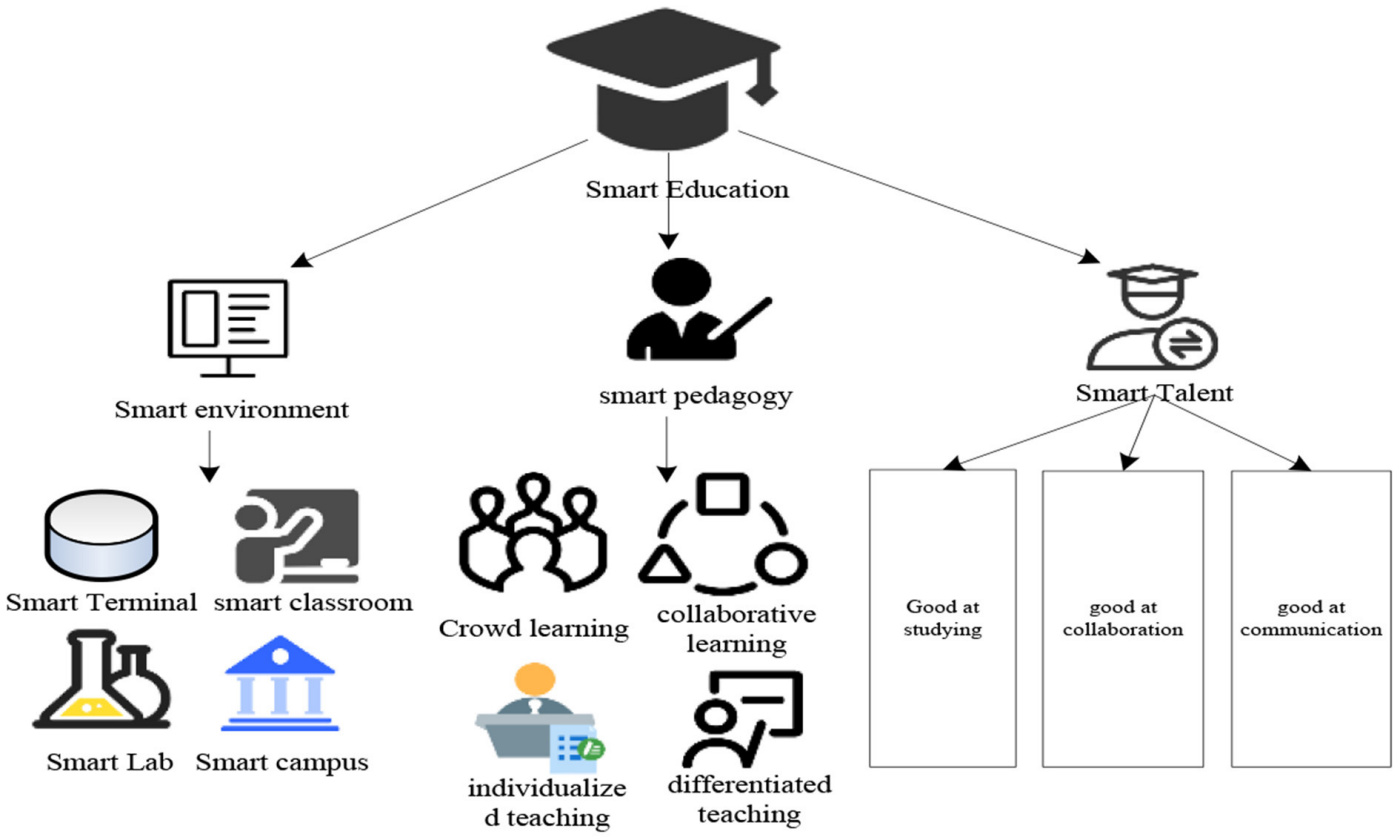
Smart education model.

With the explosive growth of social knowledge, economy and technology, a new goal has been put forward for the development of contemporary education, that is, to realize the goal of modern education through educational informatization. The concept of smart education may be a shortcut to achieve this goal (13). The smart education model consists of three elements, namely the smart learning environment, the smart teaching method, and the smart learning talents, as shown in Figure 2.

As can be seen from Figure 2, from the perspective of the traditional sense, smart education is an education that develops brain wisdom and comprehensive quality by imparting systematic knowledge to learners, which enabling them to master practical skills. Viewed from the perspective of the information age, smart education is a modern education scenario with interconnection, individuality and intelligence built through modern information technology, which promotes the transformation of educational concepts and service functions in turn. In summary, The feature of modern smart education is the deep integration of modern information technology and educational activities. It is a new educational model aiming at developing students' thinking ability and problem-solving ability by creating intelligent learning scenarios. Virtual simulation technology is one of the important modern information technologies. Its simulation and simulation properties are especially suitable for building a smart educational environment required for educational modernization, which bringing more possibilities for the development of modern physical education experimental teaching. In addition, the virtual simulation experiment supplemented by modern information technology can also realize the establishment of a fast interactive path between the subjects of physical education experiment teaching. Teachers can more calmly lead and control the whole process of physical education experiment classroom and grasp the learning progress of students. And students can also spread their learning thinking and gain course knowledge in the contextualized learning space. It can be seen that the construction of sports virtual simulation experiment teaching project is inseparably linked with smart education theory, and is one of the theoretical cores of its construction (14).

According to the research conclusions of the above scholars, the virtual simulation experimental teaching has distinct essential characteristics and external advantages compared with the traditional experimental teaching, as shown in Table 1.

**Table 1:** Characteristics of traditional teaching and virtual experiment teaching.

**Content**	**Traditional experimental teaching**	**Virtual simulation experiment teaching**
Comprehensive	The experimental content is monotonous and the knowledge update speed is slow	Rich experimental content and fast knowledge update
Reversibility	The experimental process is irreversible	The experimental process can be repeated many times
Safety	Experiments are dangerous	The experimental process is safe and reliable
Exemplary	In-person action demonstration and explanation, the communication efficiency between teachers and students is not high	Multi-angle action demonstration and explanation, real-time communication efficiency
Ease of use	The experimental equipment is limited by physical space, and the sharing is poor	The experimental equipment is not limited by physical space, and the sharing is good
Inspiring	The learning process is relatively boring, and the learning interest is low	The learning process is interesting and the learning interest is high
Cost-effective	High cost of investment and maintenance of cutting-edge instruments	One-time investment, low maintenance cost

The application of virtual simulation technology in college sports special courses can break through the limitations of traditional classroom teaching methods and their teaching methods. In the application of sports training, some scholars believed that virtualized simulation training can greatly avoid the movement risks of difficult and complex technical movements, and at the same time, it can simulate, deconstruct and analyze movements. In this way, it can promote the scientific, effective and safe implementation of the athletes' training process, and improve the teaching technology level of the coaches in a certain extent, thereby further improving the safety, systematicness and scientificity of sports training in Chinese universities.

### Kinect algorithm

#### Kinect calibration

**Figure 3:**
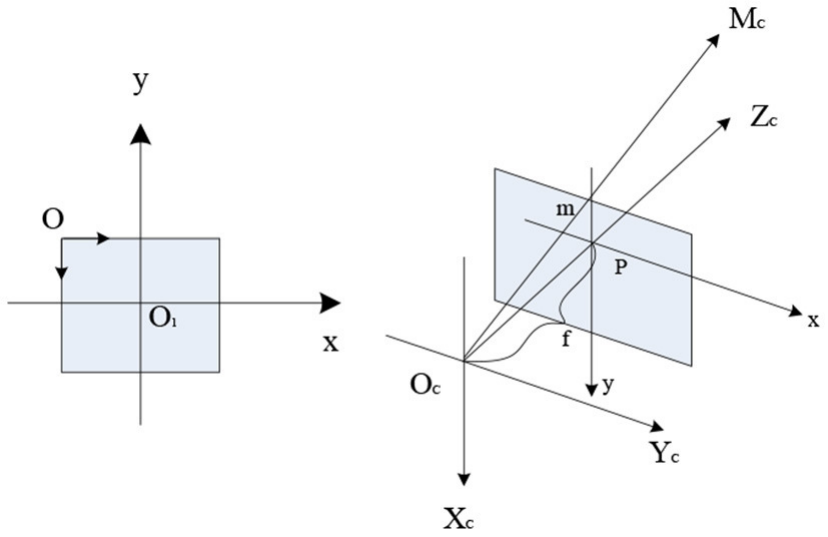
Representation of each coordinate system.

In the camera calibration model, there are three coordinate systems of mutual conversion, namely the image coordinate system, the camera coordinate system, and the world coordinate system, as shown in Figure 3.

As can be seen from Figure 3, the image coordinate system is the coordinate system (u, v) with the upper left of the image as the origin (this coordinate is in pixels), and the image physical coordinate system (x, y) (This coordinate system is in millimeters). The camera coordinate system (*x*_*c*_, *y*_*c*_, *z*_*c*_) takes the optical center *O*_*c*_ of the camera as the origin, and the distance from the optical center to the plane π is the camera focal length *f*, and the *z*_*c*_ axis coincides with the optical axis and is perpendicular to the imaging plane. By taking the photographing direction as the positive direction, *x*_*c*_and Yc are parallel to the x and y axes of the image physical coordinate system. The intersection of the ray passing through the space point M with *O*_*c*_ as the endpoint and the plane π is the point M. The image on plane π is m; the world coordinate system is (*x*_*w*_, *y*_*w*_, *z*_*w*_). Since the camera can be placed anywhere in the environment, a reference coordinate system is also required in the real-world environment to describe the position between the object and the camera. That is, the world coordinate system is used to describe the positional relationship between the object and the camera, which conforms to the right-hand rule (11).

The numerical conversion relationship between the image coordinate system and the physical coordinate system is:


(1) $$\begin{array}{l} \left[\begin{array}{c} u\\ v\\ 1 \end{array}\right]=\left[\begin{array}{ccc} \frac{1}{d_{x}} & 0 & c_{x}\\ 0 & \frac{1}{d_{y}} & c_{y}\\ 0 & 0 & 1 \end{array}\right]\left[\begin{array}{c} x\\ y\\ 1 \end{array}\right] \end{array}$$

*d*_*x*_, *d*_*y*_, *c*_*x*_, *c*_*y*_ contained in the above Formula are commonly used four-parameter camera models. After the transformation, it can be obtained:


(2) $$\begin{array}{l} \left[\begin{array}{c} u\\ v\\ 1 \end{array}\right]=\left[\begin{array}{ccc} d_{x} & 0 & -c_{x}d_{x}\\ 0 & d_{y} & c_{y}d_{y}\\ 0 & 0 & 1 \end{array}\right]\left[\begin{array}{c} x\\ y\\ 1 \end{array}\right] \end{array}$$

The conversion relationship between point (*x, y*) in the image physical coordinate system and camera coordinate system (*x*_*c*_, *z*_*c*_) is:


(3) $$\begin{array}{lll} x & = & f_{x}\frac{X_{c}}{Z_{c}} \end{array}$$

(4) $$\begin{array}{lll} y & = & f_{y}\frac{Y_{c}}{Z_{c}} \end{array}$$

Among them, *f*_*x*_, *f*_*y*_ is the focal length of the camera. The positional relationship between the camera coordinate system and the world coordinate system can be represented by a rotation matrix R and a translation vector t. Its conversion relationship is:


(5) $$\begin{array}{l} \left[\begin{array}{c} X_{c}\\ Y_{c}\\ Z_{c}\\ 1 \end{array}\right]=\left[\begin{array}{cc} R & t\\ 0^{\tau } & 1 \end{array}\right]\left[\begin{array}{c} X_{w}\\ Y_{w}\\ Z_{w}\\ 1 \end{array}\right] \end{array}$$

R in the Formula is a 3 × 3 orthogonal identity matrix, and t is a 3 × 1 translation vector. The camera model is the basis of the camera calibration algorithm. The parameters that need to be calibrated for a completed camera model are divided into intrinsic parameters and extrinsic parameters (15). The intrinsic parameter represents the relative relationship between the outdoor scene and the image point, and its value is fixed. The extrinsic parameters will change with the position of the camera itself. The camera model parameter table is detailed in Table 2.

**Table 2:** Camera model parameters.

Intrinsic parameters	X, Y axis focal lengtd	*f*_*x*_, *f*_*y*_
	Image center coordinates	*c*_*x*_, *c*_*y*_
Extrinsic parameters	Translation vector	t
	Rotation matrix	R

The Formula can be derived in the camera model:


(6) $$\begin{array}{lll} \frac{x}{f} & = & \frac{X_{c}}{Z_{c}} \end{array}$$

(7) $$\begin{array}{lll} \frac{y}{f_{y}} & = & \frac{Y_{C}}{Z_{C}} \end{array}$$

In matrix form, it can be expressed as:


(8) $$\begin{array}{l} Z_{c}\left[\begin{array}{c} x\\ y\\ 1 \end{array}\right]=\left[\begin{array}{ccc} f_{x} & 0 & 0\\ 0 & f_{y} & 0\\ 0 & 1 & 0 \end{array}\right]\left[\begin{array}{c} X_{c}\\ Y_{c}\\ Z_{c}\\ 1 \end{array}\right] \end{array}$$

Then by using Formulas (4), (5) and (6), the basic Formula of the calibration algorithm can be obtained:


(9) $$\begin{array}{l} s\left[\begin{array}{c} u\\ v\\ 1 \end{array}\right]=\left[\begin{array}{ccc} f_{x} & 0 & c_{x}\\ 0 & f_{y} & c_{y}\\ 0 & 0 & 1 \end{array}\right]\left[Rt\right] \end{array}$$

It can be seen from Formula (9) that the camera calibration only needs to be performed through the plane model. The method mainly determines the internal parameter matrix of the camera by acquiring multiple images of the plane model captured by the camera at different angles and checking the pixels on the image plane.

#### Depth image inpainting based on bilateral filtering

Bilateral filtering is mainly an improvement for Gaussian filtering, and considers the influence of distance and pixel value on filtering. The product of the Gaussian function and the image pixel value information is used as the filter weight coefficient, and the optimized weight coefficient is then convolved with the image information (16). In bilateral filtering, the relationship between the output pixel value g(i,j) and the domain pixel value f(k,I) is:


(10) $$\begin{array}{l} g\left(i,j\right)=\frac{\sum_{k,f}f\left(k,l\right)w\left(i,j,k,l\right)}{\sum_{j,k}w\left(i,j,k,l\right)} \end{array}$$

In the Formula, the weight coefficient w(i,j,k,l) depends on the spatial domain kernel:


(11) $$\begin{array}{l} d\left(i,j,l,k\right)=e^{-\frac{{\left(i-k\right)}^{2}+{\left(j-k\right)}^{2}}{2\sigma^{2}}} \end{array}$$

And the range kernel is:


(12) $$\begin{array}{l} r\left(r,j,k,l\right)=e^{-\frac{f\left(i,j\right)-f\left(k,l\right)}{2\sigma^{2}}} \end{array}$$

The product is:


(13) $$\begin{array}{l} w\left(r,j,k,l\right)=e^{{}^{\frac{f\left(i,j\right)-f\left(k,l\right)}{2\sigma^{2}}+-{\frac{{\left(i-k\right)}^{2}+{\left(j-k\right)}^{2}}{2\sigma^{2}}}^{}}} \end{array}$$

When using bilateral filtering to process images, not only the geometrical proximity but also the similarity of pixel values are considered. Through the nonlinear combination of the two, the image is obtained after adaptive filtering. In this way, the edge information of the image can be well preserved while filtering out noise. Therefore, it is most suitable to use the deep image inpainting technology based on bilateral filtering to design the fitness module in the virtual sports simulation experiment mode. The reasons are that it can make the edge of the image smoother and make students more visual.

### Mean shift image segmentation algorithm

The Mean Shift algorithm has good stability and high noise immunity in practical applications. It is widely used in pattern recognition, image segmentation and filtering, and video tracking. Generally, the average value of the local kernel function centered on each data point is defined as the probability kernel density estimate for that data point. The multidimensional kernel density of sample x in a d-dimensional hypercube is estimated as:


(14) $$\begin{array}{l} f\left(x\right)=\frac{1}{nh^{4}}\overset{m}{\underset{i=1}{\sum }}k\left(\frac{x-x_{i}}{h}\right) \end{array}$$

h represents an edge of the cube, and K(x) is the kernel function. The commonly used kernel functions are:


(15) $$k_{e}(x)=\{\begin{array}{cc} \frac{C_{d}^{-1}(d+2)(1-||x||)}{2}, & ||x||<1\\ 0, & ||x||<1 \end{array}$$

(16) $$k_{g}(x)=\{\begin{array}{cc} 2\pi^{\frac{d}{2}}e^{-\frac{1}{2}||x|{|}^{2}}, & ||x||<1\\ 0, & ||x||<1 \end{array}$$

K(x) and K(x) are Gaussian and Epanechnikov kernels. If there is a constant C such that the integral of K(x) is 1, the above two Formulas can be written as:


(17) $$\begin{array}{l} K\left(x\right)=C_{d,k}k\left({\left||x|\right|}^{2}\right) \end{array}$$

Formula (14) can be rewritten as:


(18) $$\begin{array}{l} f_{h,k}\left(x\right)=\frac{C_{d,k}}{nh^{d}}\overset{n}{\underset{i=1}{\sum }}k\left({\left||\frac{x-x_{i}}{h}|\right|}^{2}\right) \end{array}$$

In the Formula, n is the number of reference samples, h is the bandwidth, and x is the ith sample point in the window, x is the data point to be processed. The bandwidth parameter h determines the size of the window, that is, the size of the local neighborhood. The choice of bandwidth can be globally fixed or adaptive. The bandwidth can be regarded as the segmentation resolution. The larger the bandwidth, the more blurred the image is. After using Formula (18) to estimate the probability density of sample x, the gradient of its probability density is:


(19) $$\begin{array}{l} f_{h,k}\left(x\right)=\Delta f_{h,k}\left(x\right)=\frac{2C_{d,k}}{nh^{d+2}}\overset{n}{\underset{i=1}{\sum }}k\left(x-x_{i}\right)\left({\left||\frac{x-x_{i}}{h}|\right|}^{2}\right) \end{array}$$

By combining Formula (19), it can be deduced that:


(20) $$\begin{array}{l} M_{h,g}\left(x\right)=\frac{1}{2C_{d,k}}h^{2}C_{d,g}\frac{\hat{\nabla }f_{h,k}\left(x\right)}{{\hat{f}}_{h,g}\left(x\right)} \end{array}$$

**Figure 4:**
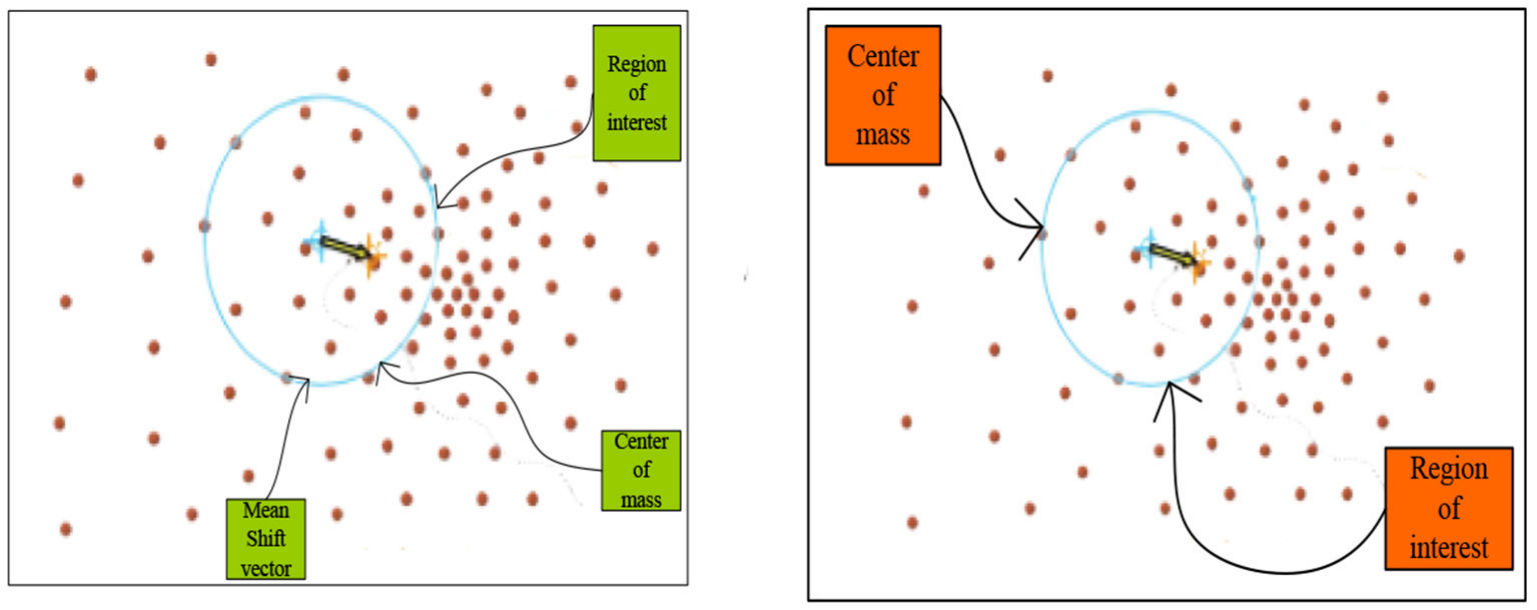
Demonstration of the mean shift method.

Mean Shift is actually a pattern search method. It searches for the next translation point in the image space while setting appropriate search stop conditions. When the panning vector is less than the given value, the panning will be stopped. The specific process is shown in Figure 4.

It can be seen from Figure 4 that the yellow arrow is the Mean Shift vector. The figure shows that a region of interest is randomly selected as the starting position, and its centroid is calculated. The Mean Shift vector represents the direction of density increase, and then a new region of interest centered on the centroid is obtained, the new centroid is calculated, and the steps are repeated to obtain the final result (17).

It can be seen from the above introduction that the depth image restoration method based on bilateral filtering can be applied to the functional module design part of virtual sports simulation experiment teaching. Its simple, local and non-iterative characteristics can be applied to the scene selection part of the function module. The Mean Shift image segmentation algorithm method can be used in the investigation and analysis of students' comprehensive health quality, which uses its excellent data analysis ability and randomness to research and summarize the investigation report.

## Design of virtual sports simulation teaching mode

### Functional structure and organization of virtual sports simulation teaching mode

**Figure 5:**
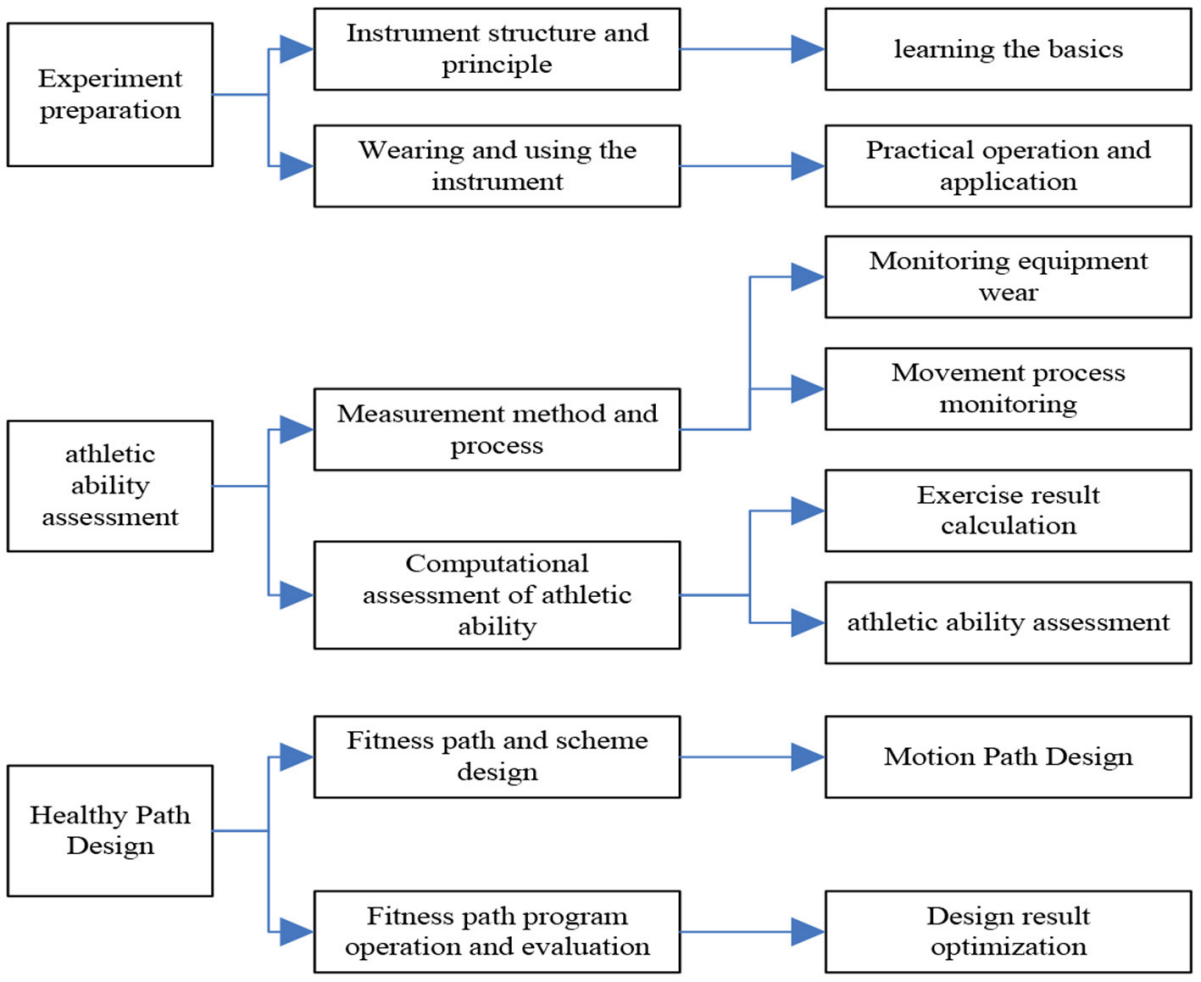
Content module of experimental teaching project.

Through the above discussion and research on the experimental objectives and experimental objectives, the top-down virtual simulation experimental structure of “Human Movement Ability Assessment and Fitness Path Design” is summarized, as shown in Figure 5.

As can be seen from Figure 5, it is divided into three modules as a whole.

#### Experiment preparation module

The instruments required in the experiment mainly include pedometer, heart rate belt, telemetry gas metabolism meter and so on. They constitute a complete set of physiological signs monitoring system to detect the subject's physiological indicators and exercise indicators to ensure the fitness and safety of exercisers. By being familiar with and mastering the working principle and application method of the whole set of sports monitoring system equipment, it can greatly help sports fitness enthusiasts to achieve fitness goals efficiently under safe conditions, which is to master the basic knowledge of human sports ability evaluation methods and processes. In this module, the parts of the instrument and equipment are disassembled, reconstructed and displayed with the help of virtual simulation technology, so that the structure and working principle of the instrument can be fully recognized by the learners, and the learners can be guided to complete the assembly and wearing of the instrument independently. And by adopting the form of observation, explanation and guidance, it helps learners to master the key points of knowledge such as the characteristics of equipment composition, working principle, the method of use and precautions, so as to strengthen the learner's mastery of the basic knowledge and promote the learner to improve the practical operation ability of the experimental equipment (18).

#### Sports ability assessment module

In the Athletic Performance Assessment module, the learning content for learners is the method and process of athletic performance assessment and the calculation and evaluation of athletic performance. The interactive operation mode of the virtual experiment can link many skills and difficulties in the exercise ability assessment experiment. And the combination of theory and operation can effectively improve learners' practical operation ability in the exercise ability assessment. Therefore, this module attaches great importance to strengthening students' practical ability and adaptability. By combining the teaching methods of theoretical guidance, control variables and independent operation, students can complete the content and process of sports ability assessment in an interactive experimental situation and deepen the knowledge and control of physiological indicators, exercise intensity, energy metabolism and evaluation process.

#### Fitness path design module

In the virtualized fitness path design, after learning the test method of exercise ability, the learners need to master the ability to provide scientific and reasonable fitness guidance strategies for fitness people with fitness goals, including exercise safety, exercise intensity and the design of fitness environment. Then the system simulates the implementation of the fitness path according to the path plan formulated by the learner to verify the safety and effectiveness of the fitness path plan. It can be showed for learners the reasons, phenomena and hazards of excessive exercise intensity and unreasonable fitness path design in a vivid interface. While cultivating safety and fitness awareness, it also provides learners with the optimal direction of program design, thereby cultivating learners' spirit of exploration and discovery (19). In addition, the teaching method of the fitness path design module consists of observation method, comparison method and independent design method. Learners can simulate real situations in the system to develop fitness coaching strategies for fitness enthusiasts. The system simulates and executes the fitness strategy according to the factors such as the path difficulty, exercise intensity and rest time designed by the learner, and intelligently evaluates and scores the strategy (20, 21). In this way, the learners' understanding of the fitness strategy formulation process and precautions and other knowledge content can be deepened.

### Functional module design

#### Experiment preparation module design

**Figure 6:**
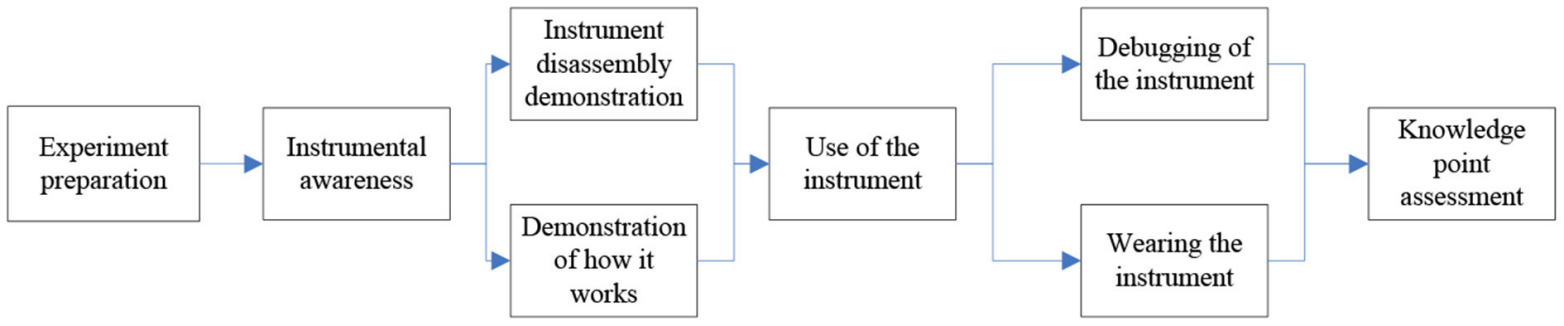
Operational steps of the experiment.

The lack of experimental equipment and instruments is one of the reasons that troubles the development of traditional experiments. Therefore, the first thing to realize the system is to understand and use the experimental instruments. Its main functions and operation steps are shown in Figure 6.

As shown in Figure 6, in the instrument cognition link of the experiment in this section, it is necessary to separately model the sports monitoring equipment and the subjects, so that they can be displayed in 360° and supplemented with text descriptions, which is easy for learners to understand and master. In the tasks of the experiment in this section, learners need to be familiar with the assembly, debugging and wearing methods of the telemetry gas metabolizer, as well as the operation interface and precautions of the heart rate monitor and pedometer, so as to lay a knowledge foundation for the next human exercise ability assessment experiment (22, 23). In this process, the system first displays the components and accessories of the equipment, so that learners have an overall impression of the equipment. Then, the system demonstrates the assembly process, debugging process and wearing method of the telemetry gas metabolism meter. With the help of virtual simulation technology, the process can be quickly restored to strengthen the learner's use and precautions for motion monitoring equipment (24). It makes the learners have a deep impression and changes the phenomenon of time-consuming, labor-consuming and consumables in the assembly of accessories in the traditional experimental process. In this process, learners need to communicate and interact repeatedly with virtual situations, so as to break the situation of unilateral indoctrination teaching and insufficient human resources of teachers in traditional experimental teaching (25, 26). It enables learners to repeatedly obtain teaching guidance in interesting and interactive teaching situations. Therefore, it is easy to master the application and operation of equipment and instruments without the constraints of time and space, which greatly improves the learner's learning motivation and efficiency.

#### Design of sports ability evaluation module

**Figure 7:**
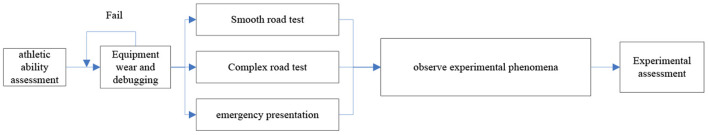
Functional module design.

The main operating place of the exercise ability evaluation experiment is carried out in a closed ideal environment with constant temperature, constant pressure and constant humidity. Power bicycles or treadmills are usually used as resistance equipment to provide exercise load, which is very different from the actual sports and fitness scenarios of the general public. Therefore, the exercise ability assessment in the experimental environment can be used as a reference, and it is of limited help for the actual exercise fitness guidance. And it is difficult to cultivate the practical ability of learners to use the knowledge of motor ability assessment after leaving the laboratory. The scene of the experiment in this section selects the outdoor road surface, and the exercise forms are the two most common fitness modes: fitness walking and running. The exercises in the gentle road section, the steep road section and the mixed road section are set respectively. The main functions and operation steps of the experiment in this section are the wearing and debugging of the equipment, the evaluation of the exercise ability of different road surfaces, and the observation of the experimental phenomenon and the recorded data changes, as shown in Figure 7.

As can be seen from Figure 7, the first part of the experiment in this section is to review the knowledge points learned in the previous section. Learners have learned to wear monitoring equipment such as telemetry gas metabolometers in the experiments in the previous section. If the operation is improper or the system is not installed in this link, the experiment will fail and the learner will re-enter this link until the learner successfully completes the debugging and wearing of the equipment. Then, according to the experimental requirements, the subjects are tested on the smooth road surface and the complex road surface respectively. This process requires the learner to observe the subject's heart rate HR, oxygen uptake *VO*_2_*MAX*, respiratory quotient RQ, respiratory rate RR and other physiological indicators at all times, and learn to calculate the energy metabolism according to the subject's weight, exercise time, displacement speed and terrain slope. The experiments in this section also set up scenarios in which abnormal movements occur, such as high heart rate, sharp drop in oxygen uptake, and abnormal breathing rate. The system alarms when the virtual subjects face safety hazards, and guides students to make further adjustments. Through this vivid virtual situational teaching, it can strengthen the learners' ability to take emergency measures in time to prevent accidental tragedies when dealing with the safety hazards of fitness practitioners. Then the experimental operation of this section is completed and the examination of the experimental content of this section is entered.

#### Design of fitness path module

**Figure 8:**
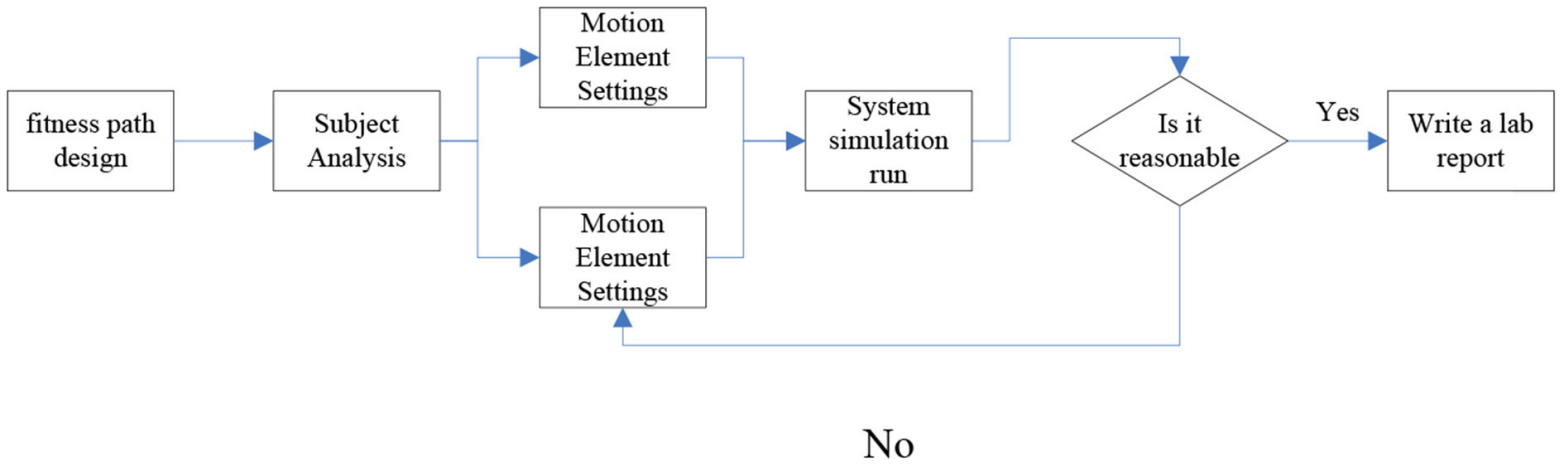
Functions and steps of the fitness path.

The fitness path design experiment expands the teaching content of the traditional exercise ability assessment experiment, deepens and strengthens the learner's ability to use engineering thinking to solve scientific problems of human movement. Its main function and operation steps are to analyze the physical condition of the bodybuilder, evaluate the exercise ability of the bodybuilder, design the fitness strategy and improve the fitness strategy, as shown in Figure 8.

According to the results in Figure 8, it can be seen that the experimental mode of the fitness path design module integrates three types of teaching methods: the task observation method, the free design method and the comparison method. The system first randomly generates the detailed information of the subject and the surrounding environmental parameters, and gives the fitness requirements. After completing the above process, the system shows the learner the fitness needs and the path design module of subjects, and asks the learner to formulate the subject's fitness path guidance strategy, and the content includes the distance of the fitness path, the slope of the path, the speed of the exercise, the time of the exercise and the time of rest and so on. After the setting is completed, the system starts to simulate and run the fitness path strategy independently designed by the learner. At this time, learners need to observe the physiological data indicators in the exercise monitoring panel. When the exercise intensity is too high or too low, the learner should intervene and adjust the exercise intensity of the subjects in time. If there is no reasonable adjustment, the system will continue to alarm and prompt the learner to continue to adjust the intensity of the exercise until the end of the experiment and finally write an experimental report according to the process and phenomenon of the experiment. This section of the experiment is to strengthen and upgrade the further application of the exercise ability evaluation experiment. Through the organic linkage of the exercise ability assessment experiment and the fitness path design experiment, the learners can strengthen the practical application ability of the exercise ability assessment. The combination of the two has changed the previous situation that the condition of the exercise ability assessment experiment is difficult to control, the experimental process is complicated, and it is difficult to carry out in a real environment. And it provides learners with personalized exploratory experimental scenarios, so that learners can truly master the method and process of sports ability assessment, and strengthen learners to use scientific and quantitative thinking mode to serve sports and fitness, so as to truly apply what they have learned.

### Health comprehensive quality learning evaluation

The evaluation system of this system uses the Mean Shift image segmentation algorithm to conduct a survey and research on a university, and adopts a combination of process evaluation and quantitative evaluation, which helps learners to review the experimental process in an objective form and realize timely interaction, error correction and feedback in the teaching process. It enables learners to clearly sort out the methods and process steps of the “Human Movement Ability Assessment and Fitness Path Design” experiment, so as to achieve the ultimate goal of “applying what you have learned” in experimental learning. The evaluation content of this system is mainly about knowledge key assessment, experimental operation assessment and innovative design of experimental report scoring methods. The scoring rules are shown in Table 3.

**Table 3:** Virtual assessment scoring rules.

**Exam topic**	**Examination content**	**Ecore**
Knowledge point	Instrument application knowledge, human motion science knowledge, safety knowledge	10
Experimental operation	Correctness of experimental steps, accuracy of data recording	30
Experimental report	Instrument debugging normative	60

It can be seen from Table 3 that the key knowledge assessment is based on multiple-choice topics. It is set in the experiment of understanding and using equipment in the first section, and is used to assess the learner's ability to master the knowledge of experimental instruments and equipment. The experimental operation assessment is set in the exercise ability assessment experimental module, and the evaluation content is carried out from three perspectives, namely the completion of the experimental steps, the correctness of data recording, and the specification of instrument debugging. The innovative design experiment report is set in the last link. The main task is to assess the rationality and effectiveness of learners' design of fitness paths and guidance strategies, which helps learners to systematically master the exercise ability assessment process and strengthens their scientific thinking and practical application in the formulation of fitness guidance strategies.

In order to make the obtained weights more scientific, more reasonable and more reliable, this paper uses AHP and Delphi method together. In the form of questionnaires, more than ten experts and scholars from a university were invited. For the last revised evaluation model in this paper and the importance level of each index involved in these models, the structure level is in descending order from important, relatively important, general, less important, and unimportant. With 9, 7, 5, 3, and 1 as the quantitative standard, the results was modified as appropriate by combining the theoretical results and suggestions of other scholars, and on the basis of knowing the scores of other experts. In this investigation and research activity, 13 of the 15 expert questionnaires were collected effectively, with an effective rate of 86.7%. After systematically and comprehensively sorting out the contents of the 13 expert questionnaires, the original data of the evaluation index weight in this paper is based on their average opinions, and the weight of each index is obtained with the help of the analytic hierarchy process and the yaahp0.60 software technology platform. The survey results are shown in Table 4.

**Table 4:** Weight results of comprehensive quality evaluation indicators for students majoring in sports training.

**Athletic training students general**	**Ideological and moral quality**	**Scientific and cultural quality**	**Physical and mental health quality**	**Practical ability innovation quality**
Quality evaluation	1.0000	1.0000	0.2000	0.2500
Ideological and moral quality	-	1.0000	0.2000	0.3333
Scientific and cultural quality	-	-	1.0000	1.0000
Physical and mental health quality	-	-	-	1.0000

It can be seen from Table 4 that the CR of the comprehensive evaluation layer is 0.0103 <0.1, and the obtained weights are valid through the consistency check. From the ranking results, “physical and mental quality” has the largest weight, reaching 0.4396. It is the focus and core of the comprehensive quality evaluation of sports training students, and it is also the most basic factor affecting the comprehensive quality of sports training students. The weight of “practical ability and innovation quality” is 0.3668, which is the evaluation index second only to “physical and mental quality” in this layer, and is an important factor for evaluating the comprehensive quality of sports training students. The weights of “scientific and cultural quality” and “ideological and moral quality” are 0.008 and 0.0928 respectively, which are of great significance for evaluating the comprehensive quality of students majoring in sports training, and are an indispensable part of the entire index system.

### Resolve of the results of health comprehensive quality evaluation

**Figure 9:**
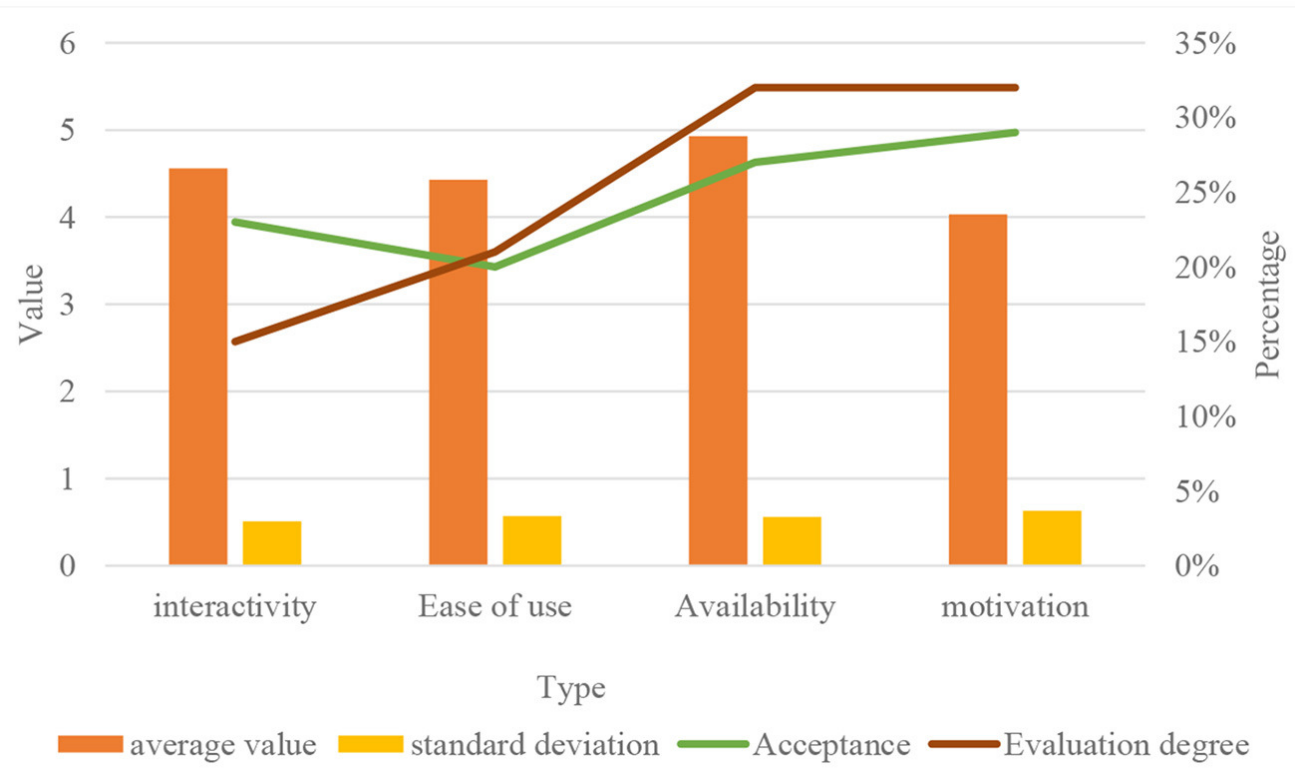
Descriptive statistics table of sports virtual simulation experiment.

The application function and teaching effect of sports virtual simulation experiment teaching are evaluated by using the Likert five-point scale, with a positive evaluation of 1–5 points. The specific results of statistical analysis are shown in Figure 9.

**Figure 10:**
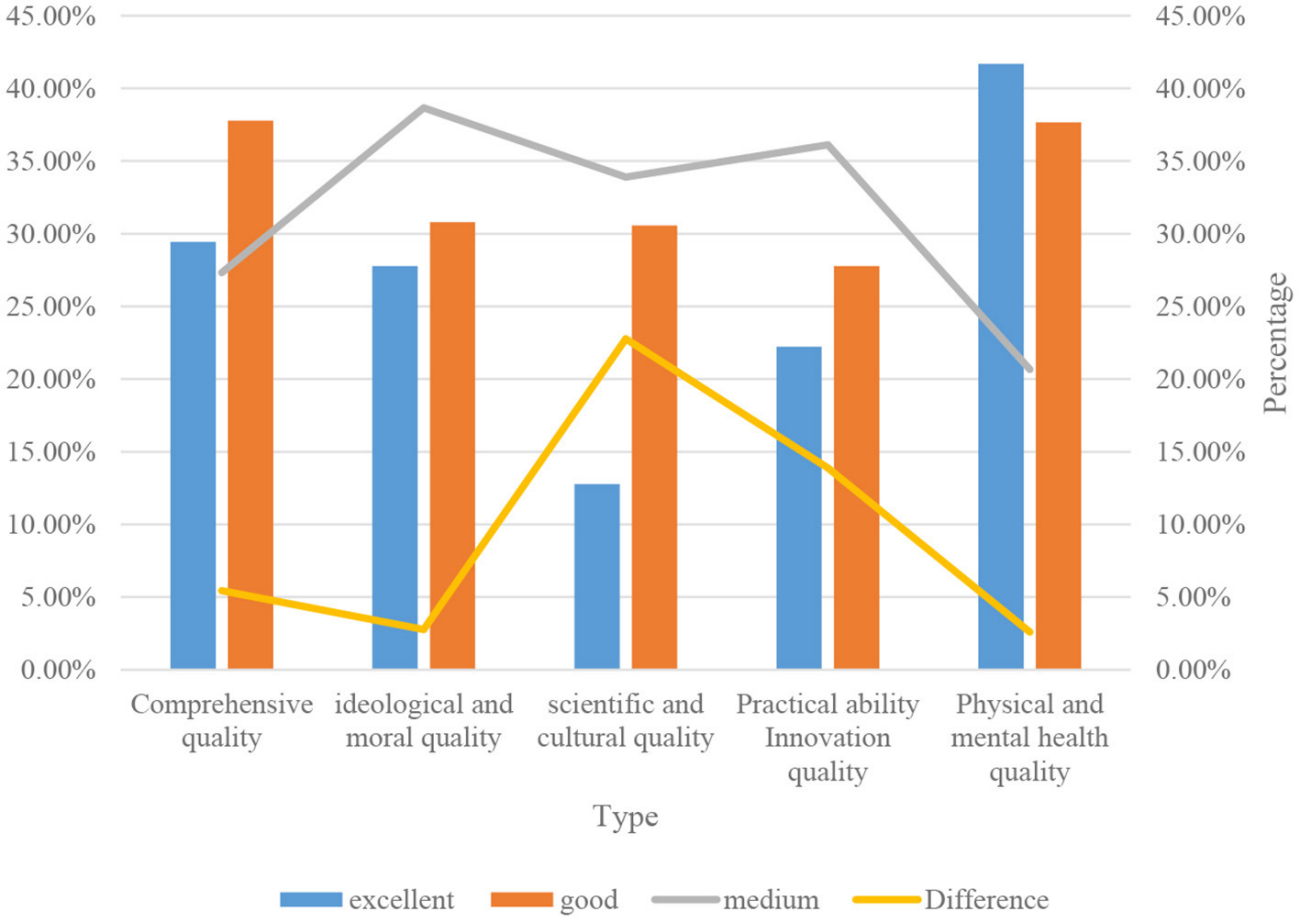
Statistical chart of student quality evaluation in a university.

According to the statistical results in Figure 9, the evaluation of a class of 30 students in a sports training major in a normal school imitated the evaluation model described above. The comprehensive quality is calculated by multiplying the evaluation matrix of the other four qualities with the first-level weight. For considering that the new evaluation system increases the content of the relevant matrix, it is required to plan related software and complete the quality evaluation of all students with the help of network and computer. According to the evaluation and analysis, the statistical analysis of the results of an efficient student quality evaluation is shown in Figure 10.

According to the evaluation results in Figure 10, it is not difficult to see the share of the indicators in the various quality grades of students in a certain college. It can be seen that the overall quality of these students has reached the level of passing or failing about 30% of the overall population. It is worth noting that the scientific and cultural quality, the proportion of good grades among all students is only 43.34%. It can be seen that the important training goal of current school research is how to use reasonable and effective methods and strategies to enhance students' scientific and cultural level, and improve students' comprehensive quality in other aspects at the same time.

At present, research on middle school students and college students (15-24-year-old youth group) mainly focuses on the investigation of the current situation of students' health literacy level and the analysis of its influencing factors. According to the health literacy status of first-year junior high school students, 17% of the respondents have health literacy. A survey on the health literacy level of some college students in Nanjing shows that only 9.8% of the respondents have health literacy. Therefore, it is urgent to publicize the comprehensive quality of health.

## Conclusions

This paper uses the virtual simulation technology in artificial intelligence and the Kinect algorithm to conduct an interventional research and analysis on the mode of physical education and the comprehensive quality of health. It systematically sorts out the research progress of virtual simulation technology in the field of education and sports. The theoretical basis and technical feasibility of the sports virtual simulation experiment teaching project are demonstrated. Physical education emphasizes the comprehensiveness of knowledge and the intersection of disciplines, and more and more needs to be integrated into the characteristics of the new era. Virtual simulation experiment has become an important application scenario of physical education teaching. The survey results show that sports virtual simulation experiment teaching has urgent and practical needs. Although it is in its infancy, its acceptance is generally high. The key to its realization strategy lies in the form of “combining virtual and real, highlighting the key points,” the method serving “highlighting sports and optimizing cognition,” and the content emphasizing “innovation-driven, comprehensive application.” However, this study still has some shortcomings. Due to the lack of experience in the design and development of the sports virtual simulation experiment teaching system, a lot of time is consumed. In addition, due to the limitations of many objective conditions, it is difficult to convene too many volunteers to participate, resulting in the problems of low sample size and relatively single analysis method in the evaluation of system application effect, which has a certain impact on the conclusions of this study. And it still needs to be strengthened in terms of fineness and reduction. It is hoped that with the continuous deepening of the follow-up research work and the feedback optimization of teaching applications, the sports virtual simulation experiment teaching system involved in this project can be improved and enhanced in terms of interactive performance, teaching scenarios and teaching effects, and finally make this system become a representative virtual simulation experiment teaching project.

## Data availability statement

The original contributions presented in the study are included in the article/supplementary material, further inquiries can be directed to the corresponding author.

## Author contributions

BZ: writing. HJ: drawing tables. XD: transferring. All authors contributed to the article and approved the submitted version.

## Conflict of interest

The authors declare that the research was conducted in the absence of any commercial or financial relationships that could be construed as a potential conflict of interest.

## Publisher's note

All claims expressed in this article are solely those of the authors and do not necessarily represent those of their affiliated organizations, or those of the publisher, the editors and the reviewers. Any product that may be evaluated in this article, or claim that may be made by its manufacturer, is not guaranteed or endorsed by the publisher.

## References

[1] Evseev S Evseeva O Aksenov A Yu V Shelekhov A. All-russian physical education and sports program “ready for labor and defense” (GTO). Hum Sport Med. (2020) 20:27–35. 10.14529/hsm20s104

[2] Silverman S. Attitude research in physical education: a review. J Teach Phys Educ. (2017) 36:303–12. 10.1123/jtpe.2017-0085

[3] Han Z. Research on sports balanced development evaluation system based on edge computing and balanced game. Sec Commun Networks. (2021) 2021:1–8. 10.1155/2021/5557138

[4] Zhao S. Research on scientific sports training of students majoring in physical education. Rev Bras Med Esporte. (2021) 27:460–3. 10.1590/1517-8692202127042021_016334574804

[5] Mandra Y Kotikova A Svetlakova E Semencova E Khodko V. Tooth hard tissues diseases of athletes: treatment and prevention features. Actual Probl Dent. (2020) 16:37–46. 10.18481/2077-7566-20-16-2-37-46

[6] Dye M Stamps DS Mason M Saria E. Toward autonomous detection of anomalous GNSS data via applied unsupervised artificial intelligence. Int J Semant Comput. (2022) 16:29–45. 10.1142/S1793351X22400025

[7] Musulin J Egota SB Štifanić D Lorencin I And̄elić N Šušteršić T. Application of artificial intelligence-based regression methods in the problem of COVID-19 spread prediction: a systematic review. Int J Environ Res Public Health. (2021) 18:1–39. 10.3390/ijerph18084287 33919496 PMC8073788

[8] Hariharan R Rahul I Darshanam MD. MPPT based on artificial intelligence system for photovoltaic system using virtual instrumentation. J Crit Rev. (2021) 7:1284–90. 10.31838/jcr.07.05.237

[9] Saini R Mussbacher G Guo JLC Kienzle J. Automated, interactive, and traceable domain modelling empowered by artificial intelligence. Softw Syst Model. (2022) 21:1015–45. 10.1007/s10270-021-00942-6

[10] Bagaria V Tiwari A. Augmented Intelligence in Joint Replacement Surgery: How can artificial intelligence (AI) bridge the gap between the man and the machine? Arthroplasty. (2022) 4:1–4. 10.1186/s42836-021-00108-1 35236504 PMC8808959

[11] Lu H Li Y Min C Chen X. Brain intelligence: go beyond artificial intelligence. Mob Netw Appl. (2017) 23:368–75. 10.1007/s11036-017-0932-8

[12] Liu R Yang B Zio E Chen X. Artificial intelligence for fault diagnosis of rotating machinery: a review. Mech Syst Signal Process. (2018) 108:33–47. 10.1016/j.ymssp.2018.02.01633267123

[13] Bose BK. Artificial intelligence techniques in smart grid and renewable energy systems—some example applications. Proc IEEE. (2017) 105:2262–73. 10.1109/JPROC.2017.2756596

[14] Lourdes VG. New challenges for ethics: the social impact of posthumanism, robots, and artificial intelligence. J Healthc Eng. (2021) 2021:1–8. 10.1155/2021/5593467 34194684 PMC8203357

[15] Palomino R Low KB Ji C Petrovic I Schmitz T. Micro computed tomography analysis of four-way conversion catalysts using artificial intelligence-enabled image processing. Microsc Microanal. (2021) 27:1028–9. 10.1017/S1431927621003883

[16] Ladd AM Diehl DL. Artificial intelligence for early detection of pancreatic adenocarcinoma: the future is promising. World J Gastroenterol. (2021) 27:1283–95. 10.3748/wjg.v27.i13.1283 33833482 PMC8015296

[17] Freitas C. Inteligncia artificial na anlise de vida til de baterias / artificial intelligence in battery life analysis. Braz J Dev. (2021) 7:24215–33. 10.34117/bjdv7n3-227

[18] Ong J Selvam A Chhablani J. Artificial intelligence in ophthalmology: Optimization of machine learning for ophthalmic care and research. Clin Exp Ophthalmol. (2021) 49:413–5. 10.1111/ceo.13952 34279854

[19] Tan TE Wong TY Hons D. Artificial intelligence for prediction of anti–VEGF treatment burden in retinal diseases: towards precision medicine. Ophthalmol Retina. (2021) 5:601–3. 10.1016/j.oret.2021.05.001 34243967

[20] Wang Q Lu P. Research on application of artificial intelligence in computer network technology. Int J Pattern Recognit Artif Intell. (2019) 33:1959015. 10.1142/S0218001419590158

[21] Qiao L Li Y Chen D Serikawa S Guizani M Lv Z. survey on 5G/6G, AI, and Robotics. Comput Elect Eng. (2021) 95:107372. 10.1016/j.compeleceng.2021.107372

[22] Yang X Li H Ni L Li T. Application of artificial intelligence in precision marketing. J Org End User Comput. (2021) 33:209–19. 10.4018/JOEUC.20210701.oa10

[23] Ogudo KA Muwawa Jean Nestor D Ibrahim Khalaf O Daei Kasmaei H. A device performance and data analytics concept for smartphones' IoT services and machine-type communication in cellular networks. Symmetry. (2019) 11:593–609. 10.3390/sym11040593

[24] Sulaiman N Abdulsahib G Khalaf O Mohammed MN. Effect of Using Different Propagations of OLSR and DSDV Routing Protocols. In: Proceedings of the IEEE International Conference on Intelligent Systems Structureing and Simulation. p. 540–5. (2014).

[25] Wang G Zhou J. Dynamic robot path planning system using neural network. J Intell Fuzzy Syst. (2021) 40:3055–63. 10.3233/JIFS-189344

[26] Song H Fink GA Jeschke S. Security and Privacy in Cyber-Physical Systems: Foundations, Principles and Applications. Chichester, UK: Wiley-IEEE Press. (2017).

